# Nanomaterial-based biosensors: a new frontier in plant pathogen detection and plant disease management

**DOI:** 10.3389/fbioe.2025.1570318

**Published:** 2025-04-23

**Authors:** Jeetu Narware, Jharjhari Chakma, Satyendra P. Singh, Divya Raj Prasad, Jhumishree Meher, Prachi Singh, Priya Bhargava, Shraddha Bhaskar Sawant, Pitambara, Jyoti P. Singh, Nazia Manzar, Abhijeet Shankar Kashyap

**Affiliations:** ^1^ Department of Mycology and Plant Pathology, Institute of Agriculture Sciences, Banaras Hindu University, Varanasi, Uttar Pradesh, India; ^2^ Department of Kayachikitsa, Faculty Ayurveda, Institute of Medical sciences, Banaras Hindu University, Varanasi, Uttar Pradesh, India; ^3^ Bihar Agricultural University, Bhagalpur, Bihar, India; ^4^ ICAR-National Bureau of Agriculturally Important Microorganisms, Mau, Uttar Pradesh, India

**Keywords:** nanobiosensor, nanotechnology, detection, plant pathogen, disease

## Abstract

Nanotechnology has significantly advanced the detection of plant diseases by introducing nano-inspired biosensors that offer distinct advantages over traditional diagnostic methods. These biosensors, enhanced with novel nanomaterials, exhibit increased sensitivity, catalytic activity, and faster response times, resulting in improved diagnostic efficiency. The increasing impact of climate-induced stress and emerging plant pathogens have created an urgent demand for real-time monitoring systems in agriculture. Nanobiosensors are revolutionizing plant disease management by enabling on-site detection of pests and weeds, facilitating precise pesticide applications. This article provides a comprehensive overview of the development and application of nanobiosensors in real-time plant disease diagnosis. It highlights key innovations, such as smartphone-integrated nanozyme biosensing and lab-on-a-chip technologies. Special emphasis is placed on the detection of molecular biomarkers, demonstrating the critical role of nanobiosensors in addressing the evolving challenges of plant disease management and agricultural sustainability.

## 1 Introduction

A biosensor is a tool used to detect biomarkers with sensitivity and selectivity, providing benefits compared to traditional diagnostic methods. Detecting a multitude of diseases with biosensors requires exceptionally precise disease-associated biomarkers, a minimally invasive or non-invasive approach, and meticulous checks to differentiate among markers linked to various health conditions. The importance of biosensors in disease detection lies in their capacity for promptly identifying disease onset, monitoring overall health, and facilitating rapid interventions for affected individuals (Mahapatra and Chandra, 2020). Fluorescence-based nanobiosensors have emerged due to recent progress, serving various medical purposes. There is research focused on employing biosensors to detect conditions such as cardiovascular diseases, cancer, and diabetes (Gouvea, 2011). Nanotechnology has been instrumental in introducing a groundbreaking type of biosensor known as the nanobiosensor. These biosensors have proven highly effective in numerous modern research fields, including environmental studies (Mahmoudpour et al., 2019), cell physiology (Shi et al., 2013), clinical detection (Mahapatra and Chandra, 2020; Mahato et al., 2018; Shetti et al., 2020), and to examine the space consequences on astronauts (Roda et al., 2018; Roda et al., 2020). Biosensors consist of three key components: (a) a biorecognition element (BRE), (b) a transducer, and (c) an amplifier and processor. A nanobiosensor is a small-scale apparatus that employs magnetic, optical, or electronic methodologies within a tiny sensor to analyze biological or biochemical occurrences (Shi et al., 2013; Roda et al., 2018; Roda et al., 2020; Di Giusto et al., 2005).

**FIGURE 1:**
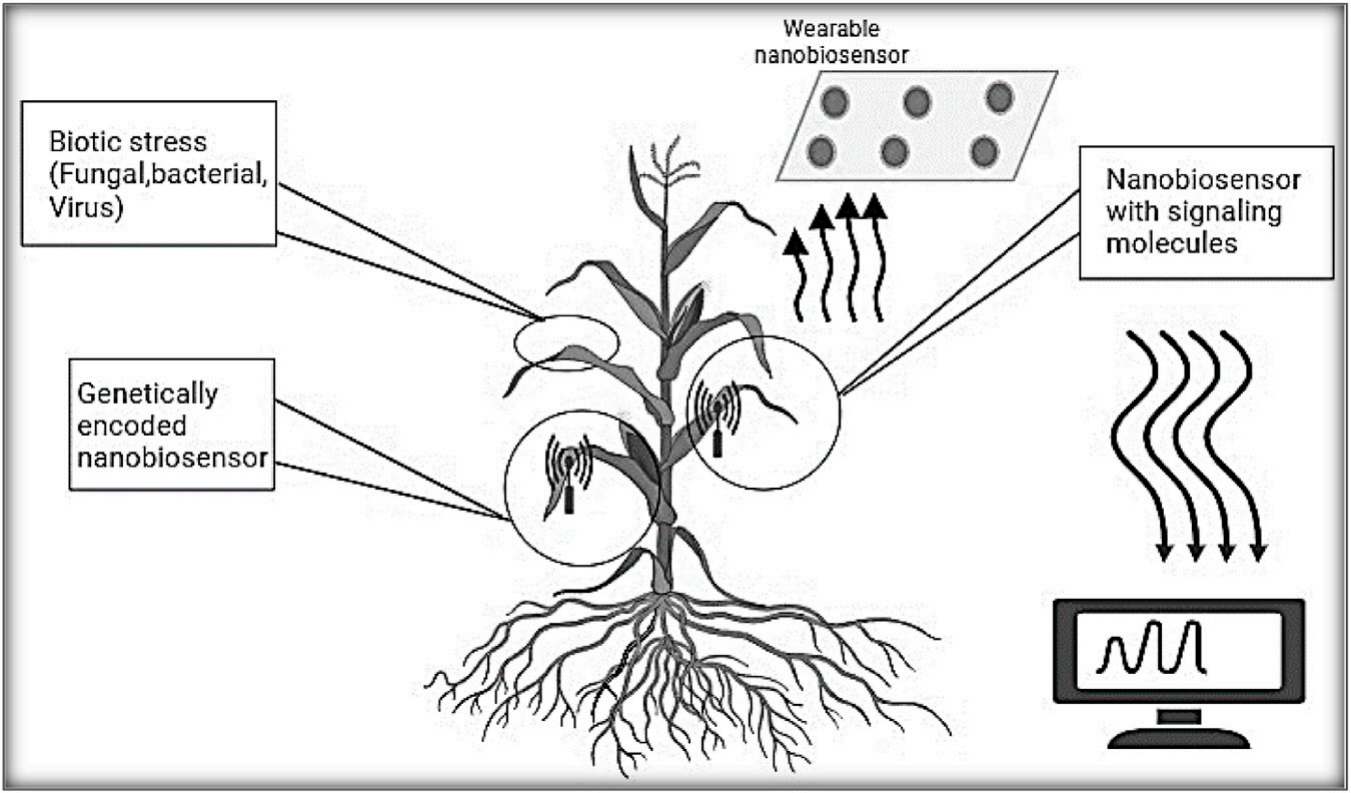
Nanomaterial-based sensors for detection plant diseases by monitoring bacterial, fungal and viral plant pathogens.

Nanobiosensors are the result of interdisciplinary research, drawing from fields like nanotechnology, biology, chemistry, and medical science (Giraldo et al., 2019; Roda et al., 2020). In most cases, nanobiosensors entail linking a biological recognition element onto a signal transducer’s surface. This initiates a heterogeneous reaction between the biorecognition element and the analyte, highlighting the pivotal importance of biosensing interface design in nanobiosensor advancement. The current progress in nanobiotechnology and advanced electronics fabrication technology has converged, giving rise to a novel category of biosensors known as nanobiosensors. These progressions represent a modern phase in nanobiotechnology, notably in the realm of diagnosing plant illnesses. Figure 1 demonstrates the utilization of nanobiosensors for detecting plant pathogens. The utilization of functionalized (bio) sensors, operating through diverse transduction processes and integrated with nanomaterial-based structures, holds the potential to establish distinct associations with a wide range of substances linked to plant diseases. These devices based on nanomaterials seem to offer promising alternatives to the conventional, more extensive methods of pathogen detection. Despite several recent articles discussing the application of nanotechnology in smart plant sensing, there remains a substantial amount of work to be undertaken in this field.

It's crucial to detect plant pathogens prior to the onset of disease symptoms in order to proactively monitor plant wellbeing and devise an informed disease management strategy. It is crucial to differentiate between causative species because many fungal infections elicit similar changes in plants as the disease progresses. Various direct and indirect methods are employed in the detection and diagnosis of plant diseases (Feng et al., 2015; Martinelli et al., 2015). Direct methods for examining plant pathogens and biomolecular markers, like nucleic acids, proteins, and carbohydrates, involve analyzing infected plant tissues. On the other hand, indirect methods detect plant diseases by observing changes in parameters such as emissions of volatile organic compounds, as well as alterations in physiological or histological characteristics like leaf surface temperature or humidity, spectroscopic attributes of plant tissues, morphology, and growth rate (Li et al., 2020). Both direct and indirect detection methods encompass a broad spectrum of technologies, including spectroscopic, electrochemical (Valekunja et al., 2016), and molecular approaches (Verosloff et al., 2019).

**FIGURE 2:**
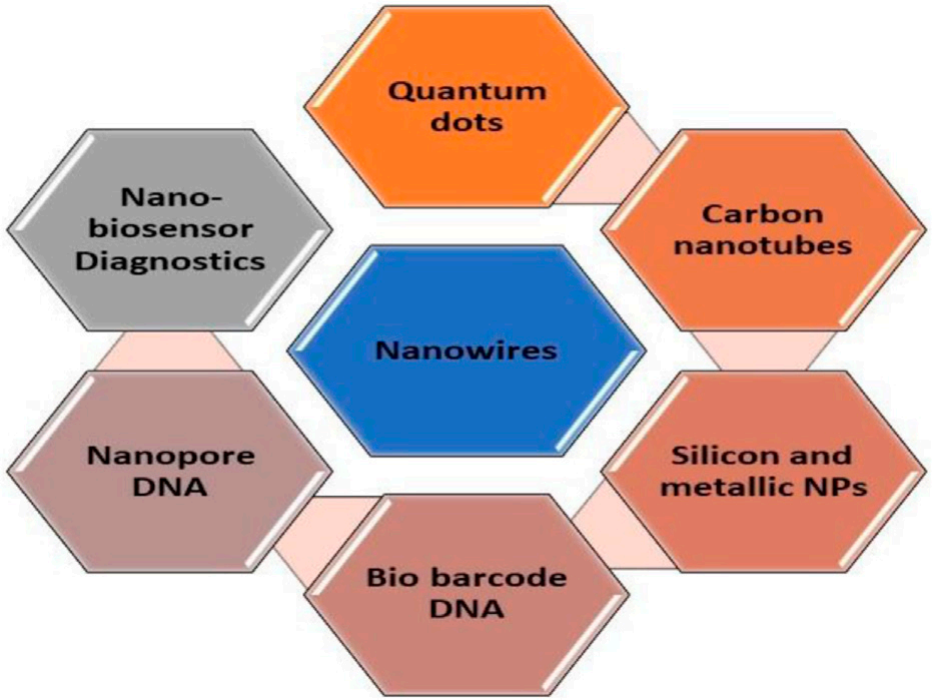
List of nanotechnology-based materials used for biosensing.

Recent endeavors have primarily focused on advancing early pathogen detection technologies to enhance sensitivity, precision, and detection speed. These efforts encompass three categories of molecular assays: enzyme-linked immunosorbent assay (ELISA), polymerase chain reaction (PCR) testing, and loop-mediated isothermal amplification (LAMP) assay, all of which rely on protein-based or nucleic acid technologies. ELISA is recognized as a highly developed serology-based diagnostic method for fungal pathogens, allowing for pathogen identification via a colorimetric reaction visible without the aid of magnification. While the classic ELISA method has become the established approach for diagnosing various pathogens across different domains such as environmental, chemical, biotechnological, health, and agricultural analyses, it continues to exhibit certain limitations due to its limited sensitivity and accuracy. Despite improvements in targeting specific pathogens with increased accuracy and precision, these commonly employed techniques still present some drawbacks. Among these challenges are extended diagnostic timelines, complicated sample preparation steps, the necessity of transporting samples from field sites to specialized labs, and a dependence on skilled professionals (Dyussembayev et al., 2021). Recent initiatives have concentrated on merging DNA and immunological techniques while incorporating various nanomaterials like silica, metallic nanoparticles, nanowires, carbon tubes, quantum dots (QDs), bio barcode DNA, and other nanomaterials (Cardoso et al., 2021). This integration has led to the development of systems like nanoparticle-based biosensors (Figure 2), which facilitate the visual detection of disease-causing agents with economic significance (Zhan et al., 2018). Some of the primary nanoscale instruments utilized in agricultural diagnostics include microneedle patches, nanopore sequencing platforms, plant wearables, and nanoparticle or array-based sensors, which can be employed in both direct and indirect methods.

## 2 Detection of plant diseases using portable sensors

A multitude of sensors, crafted with versatility in mind for applications including environmental tracking and medical analysis, have been developed and made available for commercial use. These sensors can detect analytes through a variety of signal modes, including electrical, chemical, electrochemical, optical, magnetic, or vibrational, depending on the sensor’s operating mode. Incorporating nanomaterial matrices as transducers can enhance the detection limit, while the use of bio-recognition elements like DNA, antibodies, and enzymes can enhance accuracy.

### 2.1 Biosensor platforms based on nanomaterials

#### 2.1.1 Quantum dots

Quantum dots, also known as QDs, are semiconductor nanocrystals distinguished by their distinctive photophysical characteristics, granting them remarkable potential as optical nanoprobes (Gao et al., 2018).

Quantum dots have shown effectiveness as biosensors in imaging plants and detecting diseases (Wang et al., 2020). The first recorded instance of single-celled yeast generating cadmium sulfide (CdS) crystals when exposed to cadmium salt stress occurred within the framework of semiconductor nanomaterial synthesis (Dameron et al., 1989). Their miniature dimensions (1–10 nm) enable swift uptake and transportation by plants, facilitating easy detection and tracking of their fluorescent signals within biological systems. Recently, there have been reports indicating that fungal hyphae can readily assimilate CdSe–ZnS core–shell quantum dots coated with 3-mercaptopropionic acid (Rispail et al., 2014). These nanomaterials are valuable in imaging bacteria and fungi because they have low cytotoxicity and excellent biocompatibility (Kasibabu et al., 2015). The capacity of a paper sensor based on CDs-Tb to distinguish ppGpp from structurally similar nucleotides has been demonstrated. This fluorescent Tb (III)-CD paper-based sensor can detect 3′-5′-diphosphate-5′-diphosphate, even in plants or microorganisms subjected to adverse environmental conditions (Chen et al., 2018). In the creation of such QD sensors, fluorescence resonance energy transfer (FRET) is utilized in conjunction with intrinsic fluorescence amplification or quenching. In FRET sensors, QDs act as donors, and gold NPs (Shojaei et al., 2016), organic dyes, and carbon nano dots (Shojaei et al., 2016) function as acceptors. The outcome of this energy transfer process is a decrease in the emanation of fluorescence. A recent example is the FRET-based complex sensor designed for the detection of Citrus tristeza virus. Cadmium telluride (CdTe) QDs combined with CTV coat protein (CP) and CP-labeled rhodamine dye are often used as donor–acceptor pairs for this purpose. When targeted viruses are present, CP-rhodamine is replaced by free CP, leading to the restoration of QD fluorescence (Li et al., 2020). Several types of plant viruses, such as tomato ringspot virus, bean pod mottle virus, and Arabis mosaic virus, have been identified utilizing diverse methodologies. Examples include the utilization of Fe_3_O_4_/SiO_2_ magnetic nanoparticles, SiO2/up-conversion nanoparticles at the interface, and labeled antibodies, achieving a limit of detection (LOD) of 100 ng mL^−1^. In a recent study, a nanobiosensor based on quantum dots (QDs) was highly sensitive in detecting *Candidatus Phytoplasma aurantifolia* in damaged lime plants.

Additionally, a rapid diagnostic biosensor, utilizing CdTe QDs encapsulated with specific antibodies against the *Polymyxa betae*-specific glutathione S-transferase protein, was employed for efficient evaluation of plant samples, providing accurate results within 30 min (Safarpour et al., 2012). A new optical DNA biosensor utilizing quantum dots (QDs) and employing fluorescence resonance energy transfer (FRET) has been devised for discerning specific DNA sequences in *Ganoderma boninense (* Bakhori et al., 2013). This advancement enables the identification of analogous artificial DNA sequences of the *G. boninense* gene through the examination of FRET signals (Kashyap et al., 2019). The biosensor demonstrated an impressive limit of detection at 3.55 × 10^−9^ M, showcasing exceptional sensitivity and providing a straightforward, swift, and highly responsive approach for detecting plant diseases.

Moreover, CdS quantum dots have been biosynthesized by various microorganisms, although limited research has focused on their luminescent properties (Khiyami et al., 2014). Fungus *Fusarium oxysporum*, when treated with a combination of CdCl_2_ and SeCl_4_ at room temperature, produced highly luminescent CdSe QDs. Utilizing surface-modified quantum dots (QDs) as agricultural chemicals offers a potential avenue for managing plant diseases (Li et al., 2020). In another approach, the natural antibiotic Kasugamycin (KAS) was attached to the surface of ZnO QDs (Liang et al., 2019). The produced KAS-ZnO QDs exhibited exceptional pH-responsive properties and increased photostability. They were used in a greenhouse experiment to deliver KAS and Zn (II) species controllably, resulting in a significant reduction in bacterial fruit blotch incidence (Li et al., 2020).

Image analysis and a genetic algorithm using an Arduino program to distinguish between healthy and diseased plant leaves, such as those of pepper plants, potatoes, and tomatoes affected by late blight and leaf spot (Arya et al., 2018).

It’s worth noting that many quantum dots are composed of potentially toxic heavy metals like Pb, Zn, and Hg. Therefore, the potential hazards associated with their use should not be overlooked in field applications where diagnostic effectiveness is a primary concern (Pramanik et al., 2018; Tsoi et al., 2013).

#### 2.1.2 Sequencing platform based on nanopores

Nanopore sequencing utilizes a motor protein to guide single-stranded DNA or RNA through a nanopore, which can be either protein-based or solid-state. As the nucleotides pass through the nanopore, they generate distinct electronic signals, enabling real-time sequence identification. This method, referred to as third-generation sequencing (TGS), streamlines the examination of disease-causing genetic material (Eisenstein, 2017). In recent studies, there has been significant dependence on nanopore sequencing methodologies to aid in the identification of plant pathogens (Li et al., 2020). Many investigations have utilized nanopore sequencing platforms to identify a range of plant pathogens, including bacteria, viruses, fungi, and phytoplasma, such as *Penicillium digitatum* affecting lemons. These inquiries make use of a portable sequencing tool created by Oxford Nanopore Technologies, named MinION. The entire process can be finalized in under 2 h, with outcomes comparable to those obtained through conventional diagnostic techniques like PCR and ELISA (Chalupowicz et al., 2019). Through the integration of nanopore sequencing with comprehensive transcriptome examination, scientists have successfully identified two viral strains, namely, *Candidatus Liberibacter asiaticus* and Plum Poxvirus, in peaches within a span of 24 h (Badial et al., 2018). The exceptional genome mapping capacity of MinION has facilitated the anticipation of several plant viral types within a water yam plant, encompassing Dioscorea bacilliform virus, Yam mild mosaic virus, and Yam chlorotic necrosis virus (Filloux et al., 2018).

#### 2.1.3 Nanowire as biosensor transducer

In the twenty-first century, nanotechnology fabrication techniques have led to the creation of nanowires equipped with highly specialized, small sensors and an exceptionally miniaturized design (Ariffin et al., 2014). Surface modification of these nanowires involves treating them with an amino group solution and applying enzymes to their surfaces. The modified nanowires exhibit a high surface-area-to-volume ratio, allowing them to capture and bind to biomolecules associated with plant pathogens effectively. This specific interaction triggers measurable changes in electrical, optical, or electrochemical signals, making it possible to detect plant diseases at an early stage. Notably, these biosensors have demonstrated high efficacy in detecting viral infections such as cucumber mosaic virus (CMV) and papaya ring spot virus, both of which significantly impact crop yields. The integration of nanowire-based biosensors into portable diagnostic platforms has also expanded their applicability in real-time, on-site pathogen detection, thereby revolutionizing plant disease monitoring in precision agriculture (Ariffin et al., 2014).

#### 2.1.4 Metallic nanoparticles-based detection

Metal nanoparticles, including Au, Ag, ZnS, PbS, and CdS nanoparticles, have gained a unique position in the field of biosensing due to their high surface-to-volume ratio, straightforward manufacturing processes, and adaptability for various surface functionalization. This has resulted in enhanced specificity and sensitivity, along with faster and more convenient detection methods such as color changes and electrochemical variations, proving to be a feasible alternative to traditional enzyme tags (Thaxton et al., 2006). For example, surface plasmon resonance was utilized to detect Karnal bunt disease in wheat (*Tilletia indica*) using a nanogold-based immunosensor (Singh et al., 2010). Another method involved a specific oligonucleotide labeled with fluorescein and a 2 nm gold nanoparticle at its ends, acting as a quencher, to create a nanobioreceptor for detecting phytoplasma linked to flavescence dorée in grapevines (Firrao et al., 2005). Researchers were also able to identify the pathogenic fungus *Sclerotinia sclerotiorum* by assessing salicylic acid levels in oilseeds using an electrochemical sensor based on a gold electrode containing copper nanoparticles (Wang et al., 2010). In a study focusing on Phytophthora species detection, a helicase-dependent isothermal amplification method combined with on-chip hybridization and subsequent deposition of silver nanoparticles (AgNPs) was utilized, enabling both visual and electrical output (facilitating ocular and electrical output). Additionally, colorimetric detection methods have been employed in the identification of *Pseudomonas syringae* pathovars with the assistance of AuNP-bound probes (Vaseghi et al., 2013). The colorimetric change allowed for the detection of 24–26 bacterial isolates, highlighting the suitability of the assay for the early detection of *P. syringae* with a sensitivity of 15 ng μL^−1^ of genomic DNA (Konwarh and Sharma, 2020). In a study conducted by Yüksel et al. (2015), it was demonstrated that fluorescent dyes, radioactivity, and enzyme-induced color shifts could be utilized to monitor DNA hybridization. In this approach, two distinct capture probes were attached to specific locations on an EGNP array, one for *Phytophthora ramorum* and the other for *Phytophthora lateralis*. The target DNA was hybridized with immobilized capture probes containing 2-aminopurine (2-AP) in place of adenine on the EGNP array (Yüksel et al., 2015).

#### 2.1.5 Nanofabrication imaging

Nanotechnology holds great promise in addressing challenges related to toxicity, effective imaging duration, tissue selectivity, and signal intensity by allowing precise control and customization of the chemical and physical characteristics of contrast materials. Mesoscopic nanoparticles, typically ranging from 5 to 100 nm in diameter, provide extensive surface areas, making them suitable for attaching functional groups in various pathogen detection tests (Nie, 2013).

Scientists have utilized electron beam and photolithography methods to fabricate surface patterns resembling the features of plant leaves and their internal structures. Furthermore, nano-imaging tools have been deployed to examine how pathogens infiltrate and colonize leaf tissues (Mccandless, 2005), for instance, lithography was used on silicon wafers to fabricate a pillared surface. By observing the movement of *Colletotrichum graminicola* over this substrate, which mimicked some host plant properties, researchers identified that the fungus required slight contact (at least 4.5 μm) before forming appressoria as part of ts infection mechanism.

Nanofabrication technologies have also been employed to examine the infection mechanism and behavior of *Xylella fastidiosa*, the causal agent of Pierce’s disease in grapevine xylem. This research aims to develop disease-resistant grapevine cultivars (Meng et al., 2005). In a connected investigation, Szeghalmi and co-workers (2007) explored nanostructured platforms for surface-enhanced Raman scattering (SERS), aiming for precise detection with a spatial resolution of 1 micron. Their study involved employing SERS imaging to examine dried fungal hyphae cultivated on commercially accessible nanostructured gold-coated surfaces. This illustrated that the nanofabrication methods offer standardized and replicable substrates for conducting imaging studies of phytopathogen interactions, whether *in situ* or *in vivo*.

## 3 Plant disease detection utilizing optical sensors equipped with nanomaterials

Optical sensors present numerous benefits compared to other detection techniques, such as swift identification, simplicity in operation, and comparatively affordable expenses. These sensors are devices designed to perceive and gauge alterations in the optical properties of a substance, translating them into measurable electrical signals.Common optical sensor approaches include colorimetry, fluorescence, surface plasmon resonance (SPR), flow cytometry, lateral flow assays (LFA), chemiluminescence, and bioluminescence (Yan et al., 2018). There are different types of optical sensors and nanomaterials used for the detection of plant diseases (Table 1) M13 bacteriophages were modified to exhibit a receptor-binding protein naturally inclined towards bacteria. These phages were able to attach to gold nanoparticles (AuNPs), amplifying signals and causing a visible change in color. A color-based sensor was utilized to identify pathogens, while UV-visible spectroscopy was employed for precise measurement. This method achieved a detection limit of 10^2^ CFU mL^−1^, with a linear detection range spanning from 10^2^ to 10^6^ CFU mL^−1^. The primary optical techniques for detecting plant pathogen DNA typically involve color-based tests utilizing gold nanoparticles (AuNPs), such as lateral flow assays and aggregation assays. Furthermore, fluorescent and color-based microarrays, along with electrochemiluminescence analysis, are frequently employed for this objective. Leveraging lateral flow immunoassays (LFIAs) has presented exciting prospects for enhancing signals, including the integration of nanomaterials like nanoparticles and graphene, alongside straightforward adjustments to the platform structure, such as adopting a vertical flow configuration (Parolo et al., 2013a; Parolo et al., 2013b; Rivas et al., 2014; Morales-Narváez et al., 2015; Nunes-Pauli et al., 2015). LFIAs, in a sandwich format, have been utilized to detect various plant viruses such as *Citrus tristeza virus* (CTV) and *Potato virus X* (PVX), as well as plant-pathogenic bacteria like *Erwinia amylovora*, *Xanthomonas,* and *Pantoea stewartii*, employing AuNPs tags. For instance, in identifying *Citrus tristeza virus* (CTV) in citrus leaves and fruits, Salomone and colleagues (2004) formulated an LFIA using a conventional antibody-based sandwich setup with AuNPs as a marker. Qualitative findings exhibited sensitivity akin to that of an ELISA test, displaying strong correlation. The assay’s specificity also proved satisfactory, resulting in a 5% false positive rate. The inaugural LFIA for detecting a phytopathogen, specifically *Tobacco mosaic virus* (TMV), was documented by Tsuda (1992). Subsequently, a similar approach was developed for *Potato virus X* (PVX) detection (Drygin et al., 2012). The discernment of these assays was assessed against major potato seed viruses, including Potato virus Y (PVY), Potato virus M (PVM), and Potato virus A, with sensitivities reaching as low as 2 ng mL^−1^ for PVA (Feng et al., 2015). Employed an LFIA for swift detection of *P. stewartii* in corn seed samples. The LFIA was tested against three others phytopathogenic bacteria (*Burkholderia glumae*, *Xanthomonas oryzae*, and *Pseudomonas syringae*), and none of them showed cross-reactivity, demonstrating high selectivity. The detection limit for PSS in the test was 10^5^ colony-forming units per milliliter (cfu mL^−1^) (Razo et al., 2019). Razo and co-workers in 2019 proposed enlarging gold nanoparticles (GNPs) in order to establish a sensitive lateral flow immunoassay for *R. solanacearum* detection, they developed lateral flow test strips containing gold nanoparticles (17.4 ± 1.0 nm) as a tag and antibodies specific to *Ralstonia solanacearum* conjugated to them. Tetrachloroauric (III) anion reductions on the GNP surface resulted in a signal augmentation within test zone of the test strips, and gold nanoparticles size on the test strips increased by about 100 nm, as verified by scanning electron microscopy. The method lowers *R. solanacearum* detection limit by 33-fold to 3 × 10^4^ cells mL^–1^. Entire process, including sample prep and gold enlargement, completes in just 15 min. A novel method combines UP-APCR with AuNP-based lateral flow biosensor for direct *Phytophthora infestans* assessment (Zhan et al., 2018). This method offers quick, visible detection of *P. infestans* with high sensitivity and accuracy, achieving a low detection limit of 0.1 pg μL^−1^ within 1.5 h. It effectively identifies the pathogen in affected plant samples, showing great potential for field application due to its speed, simplicity, and reliability.Various studies in the literature have explored surface plasmon resonance (SPR) biosensors, including those using DNA probes, antibodies, and aptamers, for monitoring plant infections (Wang et al., 2004; Candresse et al., 2007; Lautner et al., 2010). Lin et al. (2014) developed a label-free SPR immunosensor using gold nanorods (AuNRs) to detect *Cymbidium mosaic virus* (CymMV) and *Odontoglossum ringspot virus* (ORSV) in orchids. They coated AuNRs with antibodies against orchid viruses to enhance sensitivity and reduce color interference, achieving detection limits of 48 pg mL^−1^ for CymMV and 42 pg mL^−1^ for ORSV. Specificity was confirmed by examining target and non-target viral antigens, and performance was evaluated by detecting signal changes due to antigen-antibody binding on the AuNRs’ surface.A new surface plasmon resonance-based immunosensor was developed for detecting *Pseudocerocospora fijiensis* fungus in leaf extract samples. This sensor used a polyclonal antibody against *P. fijiensis* bacterial cell protein HF1 immobilized on a gold chip in a lateral flow assay. It showed a linear response for HF1 from 39.1 to 122 μg mL^−1^, with a detection limit of 11.7 μg mL^−1^ and a sensitivity of 0.0021 units of reflectance per ng ml^−1^ (Luna-Moreno et al., 2019). In various plant diseases, lateral flow (LF) test strips for DNA detection have been developed, often using gold nanoparticle (AuNP)-labeled DNA probes. Zhao et al. (2014) employed a competitive DNA hybridization method to detect *Acidovorax avenae* bacterial disease in melons, achieving a low detection limit of 0.48 nM. Selectivity testing against five different plant bacterial diseases showed no cross-reaction. Wei et al. (2018) used DNA hybridization on a lateral flow platform for rapid detection of Banana bunchy top virus (BBTV) using AuNPs-DNA probes, achieving a low detection limit of 0.13 nM. The BBTV-DNA lateral flow biosensor demonstrated ten times higher sensitivity than electrophoresis and showed selectivity against other viruses like Banana streak virus (BSV) and Cucumber mosaic virus (CMV).

**TABLE 1:** Visual detectors utilizing nano components for disease identification in plan**t**.

Culture/Targets	Nano material	Identification technique	Accuracy	Biological recognition	Capability assessment	Performance metrics	Notations	References
*Xanthomonas campestris*	Gold nanoparticles (AuNPs)	Colorimetric	∼100 cells	Thioled M13KE interaction with AuNP	100% specificity in diagnosis	10^2^–10^6^ CFU	Simple, sensitive, and specific Colorimetric Detection of Bacterial Species	Peng and Chen (2019)
*Phakopsora Pachyrhizi/Soybean*	fluorescent nanoparticles	Fluorescence	2.2 ng mL^−1^	Antigen-antibody agglutination	sensitivity of 2.8 a.u.n g^-1^ mL, comparable to ELISA and PCR methods	0.0032 up to to 3.2 mg mL^−1^	Nitrocellulose membrane based fluorescent detection	Miranda et al. (2013)
*Phytophthora infestans*/Tomato	Cysteine (Cys)-functionalized gold nanoparticles (Au NPs) or nanorods (Au NRs)	Colourimetric sensor	≥95% in detection of Phytophthora infestans in both Lab and field conditions	Specific recognition of gaseous (E)-2-hexenal	LOD-0.4 ppm	Phytophthora infestans suspension (1,000–10,000 sporangia ml^−1^)	Smartphone-based platform for the detection of VOCs	Li et al. (2019b)
*Alternaria panax* Whetz/Ginseng	Gold nanoparticles (AuNPs)-streptavidin particles	Single-tube nested PCR-lateral flow biosensor assay (STNPCR-LFBA)	0.01 pg μL^−1^	Mouse anti-Fam antibody reaction with AuNPs-streptavidin particles	100 times more sensitive than the traditional PCR-LFBA	0.01 pg μL^−1^ to 1 ng μL^−1^	Rapid identification of *Alternaria panax* Whetz using STNPCR-LFBA	Wei et al. (2018)
yellow leaf curl virus/Tomato	Gold nanoparticles (AuNPs)	Colorimetric	5 ngμL^−1^	Specific DNA probe- viral DNA hybridization	eliminate the need for PCR amplification and detection equipment	0.75–200 ng/μL	Fast and sensitive detection of TYLCV genome	Razmi et al. (2019)
Leaf roll virus/Potato	AuNPs and Silver	Lateral flow Immunoassay	0.2 ng mL^−1^	Antigen-antibody agglutination	LFIA with silver enhancement was 15 times more sensitive	0.1–100 ng mL^−1^	Quick and accurate on-site primary screening control of PLRV.	
*Xanthomonas arboricola pv. Pruni*/Stone fruit crops`	carbon nanoparticles	Lateral flow Immunoassay	104 CFU mL^−1^	Antigen-antibody agglutination	100% Diagnostic specificity, 96.1% Diagnostic sensitivity	10–10^8^ CFU mL^−1^	Minimally trained users to obtain reliable results in less than 15 min	
*Phytophthora infestans*/Potato	Gold Nanoparticle (AuNPs)	Lateral flow assay	0.1 pg µL^−1^	streptavidin-biotin reaction AuNP-probe	100% diagnostic specificity	0.1 pg/μL-100 pg/μL	High amplification efficiency of the UP-APCR and the portable gold nanoparticle-based lateral flow biosensor	Zhan et al. (2018)

LOD: limit of detection; AuNPs: Gold nanoparticles; VOCs: Volatile organic compounds; LSPR: surface plasmon resonance; LFA: lateral flow assay ELISA: enzyme linked immuno sorbent assay; PCR: polymerase chain reaction.

### 3.1 Electrical and electrochemical

Electrochemical and electrical approaches for plant disease detection have gained popularity, offering advantages such as straightforward procedures, sensitivity, selectivity for specific infections, and the potential for portable commercial equipment for *in situ* measurements (Khaled et al., 2018; Ray et al., 2017; Feng et al., 2015). These methods have been employed in various environments, including greenhouses, fields, and postharvest storage containers, allowing for the detection of infections in the air, water, and on seeds. Most of these technologies rely on identifying pathogens through the use of biosensors (Martinelli et al., 2015). Functionalized electrode interaction with the analyte causes electron transfer, enabling detection and quantification via various electrochemical assays such as amperometric, voltammetric, potentiometric, and impedimetric methods (Cesewski and Johnson, 2020; Fang, 2020), for example, Freitas and colleagues utilized gold nanoparticles (AuNPs), magnetic beads, anti-CP-CTV antibodies, and the horseradish peroxidase (HRP) enzyme to detect the capsid protein from the Citrus tristeza virus (CP-CTV). To diagnose citrus canker, Haji-Hashemi and their team designed an electrochemical immunosensor for the detection of the PthA protein. They immobilized anti-PthA antibodies on AuNPs (GNP), and the antigen was detected using the fast Fourier transform square wave voltammetry (FFT-SWV) method. The FFT-SWV peak currents decreased as the PthA concentration increased due to the formation of antigen-antibody complexes. The sensor demonstrated a linear relationship between the current response and the logarithm of PthA concentration in the range of 0.03–100 nM, with a limit of detection (LOD) of 0.01 nM. The immunosensor exhibited repeatability (a relative standard deviation of 3.9%), selectivity (as determined by studies of healthy plant sap, BSA, and myoglobin), and stability (97 percent of the original response after 7 days). It was also tested with artificially affected healthy plant sap samples. The electrochemical biosensor’s results were in agreement with those obtained using the PCR approach, suggesting its potential for early detection of citrus canker disease. Utilizing differential pulse voltammetry, Fang and co-workers detected *p*-ethylguaiacol using SnO_2_ and TiO_2_ nanoparticles on screen-printed carbon electrodes (SPCE) (DPV). The analyte is a volatile generated by *Phytophthora cactorum* fungus-infected fruits and plants. Two very different sensors had a low LOD: 35 nmol L^−1^ for the TiO_2_ electrodes and 62 nmol L^−1^ for the SnO_2_ electrodes, accordingly. An interference analysis with six chemicals revealed an increased response variation of 6.7 percent, indicating the sensors’ strong selectivity. By simulating the makeup of a genuine fruit volatile signature, the determination of *p*-ethylguaiacol in real infected samples was tested. Groundnut bud necrosis tospo virus (GBNV) is a disease involved for viral epidemics that needs early identification and regular monitoring to minimize rapid vector transmission. Chaudhary and co-workers employed a graphene oxide (GO) based electrochemical immunosensor to detect it (Chaudhary et al., 2021). The ITO substrates were coated with GO to enhance electrical conductivity via sp2 carbon domains, enabling modification of the sensor with anti-GBNV antibodies.The GBNV nucleocapsid (GBNV-N) protein was detected using the DPV approach in the range of 0.5–150 ng mL^−1^ with a LOD of 5.7 0.7 ng mL^−1^. The sensor’s usage was investigated, and it was discovered that throughout three and seven cycles, activity reduced by less than 3% and 10%, respectively. Some other method for detecting plant diseases is using an electronic nose (e-nose) (Cui, et al., 2018; Cellini et al., 2017). Different types of electrical, electrochemical techniques., and nanomaterials used for the detection of the plant disease are presented in Table 2.

**TABLE 2:** Enhanced diagnosis of plant diseases: Combining electrical, electrochemical methods, and nanomaterials.

Target/Culture	Substrate	Nanomaterial	Bio-recognition	LOD or accuracy	Detection method	Other figures of merit/Minimum value to be detected	Observation	References
Citrus/Sec-delivered effector 1 (SDE1)	Gold (Au) microelectrodes onto Si/SiO2 wafer	Single-walled carbon nanotubes (SWNTs)	anti-SDE1 polyclonal antibodies (pAb)	LOD: 5 nM	Field-effect transistor (FET)/chemiresistor-based biosensors	3 nM to at least 2.6 µM	Adopting the novel detection strategy targeting the secreted protein biomarker, SDE1, addresses some of the challenges faced by current methods of nucleic acid-based assays and symptom-based diagnosis	Tran et al. (2020)
Aspergillus and Rhizopus fungi/Strawberry	ENIG (Electroless Nickel Immersion Gold)	multi-walled carbon nanotubes (MWCNTs)	Chemical Sensor	—	E-nose	—	Using a carbon nanostructure-based electronic nose system to detect fungal infections like Rhizopus sp. or Aspergillus sp. Nigri in strawberries	Greenshields et al. (2016)
Cucumber mosaic virus/Cucumber	Gold microelectrodes	Polypyrrole nanoribbon	polyclonal anti-CMV IgG	LOD 10 ng mL^−1^	chemiresistive immunosensor	Nanoimmunosensor response highly influenced by buffer concentration	detection of CMV using a chemiresistive immunosensor based on antibody-functionalized PPy nanoribbons	Chartuprayoon et al. (2013)
Phytophthora/Strawberries	p-type silicon wafer	single-walled carbon nanotubes (SWNTs)	ssDNA	0.13% saturated vapor of P-ethylphenol	E-nose	s6DNA-SWNTs relationship with the 4-ethyl phenol concentration in the range of 0.25%–20% and 20%–100%	Utilizing an FET modified with SWCNTs and ssDNA to detect 4-ethyl phenol for diagnosing P.cactorum in strawberry plants	Wang et al. (2020)
*Phytophthora infestans* infection/Tomato	kirigami-inspired stretchable substrate	graphene-based sensing materials and flexible silver nanowire electrodes	thiourea@rGO sensors and AuNP@rGO sensors	>97% accuracy	Chemi-resistive sensor array	—	diagnose tomato late blight as early as 4 days post inoculation and abiotic stresses such as mechanical damage within 1 h	Li et al. (2021)
*Xanthomonas axonopodis/Citrus*	Bamboo-like multiwall carbon nanotubes-ionic liquid nanocomposite (BCNT-IL)	Gold nanoparticles (GNPs)	anti-PthA antibody	0.1–50 nM with a detection limit of 0.028 nM	fast Fourier transform square wave voltammetry (FFT-SWV)	0.03–100 nM, sample recovery levels between 96% and 103%, or unspecified	Using a glassy carbon electrode with gold nanoparticles (GNP), carbon nanotubes (CNT), and Prussian blue (PB) to create an improved immunoassay sensor	Haji-Hashemi et al. (2018)
Plum Pox Virus (PPV)/Stone fruit trees	gold gate electrode using a sub-monolayer of Protein G	Gold and Pentacene films	Anti-Plum Pox Virus polyclonal	LOD: 180 pg mL^−1^	electrolyte-gated organic field-effect transistor (EGOFET)	from 5 ng mL to 1 to 50 μg mL^−1^/N/A	Developing a quick and accurate biosensor to detect the PPV virus in semi-purified extracts from stone fruit trees, so that labeling is not necessary	Berto et al. (2019)
Phytophthora cactorum/-	Screen-printed carbon (SP) electrodes	TiO2 and SnO2 nanoparticles	—	LOD: 35–62 nmol L− 1	CV and DPV	LOQ: 106–188 nmol L^−1^/20.8 μmol L^−1^ of pethylguaiacol	Metal oxides are a reasonable alternative to expensive electrode materials such as gold or platinum for amperometric sensor applications	Fang (2020)
*Pantoea stewartia sbusp. Stewartia* (PSS)	Glassy Carbon Electrode (GCE)	AuNPs	horseradish peroxidase (HRP)	s 7.8 × 103 cfu mL^−1^	linear sweep voltammetric (LSV) curves	Recovery levels from 90.6% to 107.5%	Because of the combination of HRP’s catalytic activity and AuNPs’ big surface area and conductivity, the LOD is low	Zhao et al. (2014)
*Ustilaginoidea virens*	Paper electrodes	Graphene oxide	ssDNA	10 fmol L^−1^	CV and LSV	Range: 10 μmol L^−1^ to 10 fmol L^−1^	GO-enhanced paper electrodes combined with ssDNA allow for the very selective and sensitive detection of rice fake smut disease	Rana et al. (2021)
*Citrus Tristeza Virus* (CTV)/Sweet orange trees	Carbon ink 8- WE SPCE	AuNPs	monoclonal antibody anti-CP-CTV	LOD: 0.3 fg mL^−1^	Amperometry	Linear range from 1.95 to 10.0 × 103 fg mL^−1^	Citrus pathogen biomarkers are identified using immunomagnetic separation in conjunction with a single-use microfluidic device	Freitas et al. (2019)

(LOD: limit of detection; VOC, Volatile organic compounds; FET, Field-effect transistor; EGOFET, Electrolyte-gated organic field-effect transistor; E-nose–Electronic nose; FFT-SWV–Fast Fourier transform square wave voltammetry; EIS, Electrochemical Impedance Spectroscopy; SPCE, Screen-printed carbon electrode; rGO, Reduced graphene oxide; AgNW, Silver nanowire; SWCNTs, Single walled carbon nanotubes; MWCNTs, Multiwalled carbon nanotubes; GCE, Glassy carbon electrode; LSV, Linear sweep voltammetry; GO, Graphene oxide; ITO, Indium-tin oxide; AuNPs, Gold nanoparticles; ssDNA, Single strain deoxyribonucleic acid; CTV, Citrs tristeza virus; CV, cyclic voltammetry; DPV, differential pulse voltammetry; 8-WE-SPCE, working electrode screen-printed carbon electrodes; PPY, Polypyrrole; Lithographically patterned nanowire electrodeposition (LPNE); GNP, gold nanoparticle; RTBV, Rice tungro bacilliform virus; RTSV, Rice tungro spherical virus).

## 4 Detection of mycotoxins by nanobased sensor

The immune-electrode was effectively employed for the detection of AFB1 within the range of 10–100 ng dL^−1^, offering a sensitivity of 0.45 μAng^−2^, with a detection limit as low as 17.90 ng dL^−1^, and a rapid response time of 60 s. The primary purpose of utilizing such nanostructures is to expedite the identification of pathogens. Various nanomaterials, including carbon nanotubes, graphene, nanowires, nanocomposites, nanostructured metal oxides, and nanoparticles, are increasingly being utilized in the detection of pathogens and mycotoxins. Microfluidic systems, which can also be employed for real-time infection diagnosis with high sensitivity, represent another type of nanostructure platform (Baeummer, 2004). These systems offer the significant advantage of detecting specific target substances in small sample volumes and within a short time frame. For the detection of *Aspergillus ochraceus* (OTA), researchers successfully co-immobilized r-IgGs and BSA using a nanoSiO2 and chitosan-based nano-biocomposite material on an ITO substrate. Their findings indicated that the BSA/r-IgGs/CH-NanoSiO2/ITO immune-electrode exhibited optimized sensing properties for OTA recognition. In another approach, horseradish peroxidase (HRP) biosensors were employed to evaluate OTA and *Penicillium viricatum* in spiked beer and roasted coffee samples without the need for pretreatment, as described in the study conducted by Alonso-Lomillo and colleagues in 2010.And *P. viricatum* in spiked beer and roasted coffee samples with no pretreatment (Alonso-Lomilloa et al., 2010). It was improved an electrochemical immunosensor by using magnetic nanoparticles to determine ultra-trace amounts of AFM1 (up to 0.01 ppb) generated by *A. flavus* with foodstuffs (Paniel et al., 2010). A ‘lab-on-chip’ technique for the rapid, sensitive, and selective detection of zearalenone generated by *Fusarium* sp. by combining an electrokinetic magnetic bead-based electrochemical immunoassay on a microfluidic chip. For the quick and sensitive measurement of zearalenone in corn silage samples, researchers developed an immunosensor that used multi-wall carbon nanotubes and a continuous-flow technology. Ansari and co-workers (2010) showed that a sol–gel Nano-ZnO film can be used to immobilize r-IgGs and BSA can be utilized to prevent irrelevant binding affinity of r-IgGs to detect OTA with a detection range of 0.006–0.01 nM/dm. The ozonation and adsorption efficacies of altered nano-diamonds for detecting aflatoxin-B1 level were investigated. Recently it was presented an ultrasensitive approach for detecting mycotoxins using STING (signal transduction by ion nano-gating) sensing, with a detection limit of 100 fg mL^−1^ (Actis et al., 2010). To make a BSA/aAFB1-CAuNP/MBA/Au immune-electrode. Cysteamine functionalized gold nanoparticles (C-AuNP) and aflatoxin B1 antibodies (aAFB1) were immobilized on a 4-mercaptobenzoic acid-based self-collected monolayer on a gold electrode (MBA/Au). AFB1 in the range of 10–100 ng L^−1^ was detected using these electrodes. A mobile equipment that can simultaneously identify numerous bacterial, fungal toxins, and pathogens in stored food was recently created (Biswal and Misra, 2020). According to these studies, nanostructured platforms appear to be a promising alternative to traditional approaches for detecting mycotoxins and infections that damage food and agricultural products.

## 5 Tracking plant diseases through the identification of volatile organic compounds (VOCs)

Plants release volatile organic compounds (VOCs) as biomarkers into their environment through various parts like leaves, flowers, roots, and other organs, which can be influenced by factors such as self-healing, exposure to stress, herbivore browsing, pathogen infection, and pest repellence. These VOCs play a crucial role in plant responses to pest damage and diseases (Hoebeke et al., 2011). Among the botanical VOCs, terpenes like cis-jasmone (CJ), -pinene, limonene, and -terpinene are commonly found (Bruinsma et al., 2009; Han et al., 2016). Gas chromatography/mass spectrometry or gas chromatography electroantennographic detectors are traditionally used to detect these VOCs (Mesquita et al., 2017; Cheung et al., 2015). However, these methods are expensive, time-consuming, and impractical for real-time monitoring of plant VOCs. Therefore, there is a need for sensors that offer high accuracy, rapid response times, and immunity to interference for real-time plant VOC detection in agricultural applications. The detection of cis-jasmone vapor, a localized surface plasmon resonance (LSPR) sensor coated with a molecularly imprinted sol–gel (MISG) can be employed. The MISG coating enhances the LSPR sensor’s selectivity and reduces sensitivity. To further enhance the sensor’s sensitivity, gold nanoparticles (AuNPs) have been embedded in the MISG. According to Shang and colleagues in 2018, sensors coated with MISG containing 20 µL of 30-nm AuNPs exhibited higher sensitivity compared to sensors coated with other films. The precise detection of gaseous (E)-2-hexenal, one of the primary VOC indicators produced during *P. infestans* infection, researchers used cysteine (Cys)-functionalized gold nanoparticles (Au NPs) or nanorods (Au NRs) as plasmonic analytical colorants in a sensor array. This portable device incorporates a disposable colorimetric sensor array consisting of plasmonic nano-colorants and chemo-responsive organic dyes, enabling the detection of essential plant volatiles at the parts per million (ppm) level within a minute (Li Y. et al., 2019).

## 6 Commercially available devices

Effective plant disease detection relies on the availability of commercial bio-recognition elements, such as antibodies, DNA probes, and aptamers. Immunoassay-based technologies are commonly employed for the detection of phytopathogens, with lateral flow devices, tissue-print ELISA, and plate-ELISA kits being among the most widely used methods. Several research studies have highlighted the use of commercial kits based on immunoassays, including a portable kit designed for orchid virus detection and the Agritest lateral flow kit for identifying *Erwinia amylovora*, the bacterial agent responsible for pome tree disease. Furthermore, a commercial diagnostic tool has been developed for detecting *xanthomonas* wilt in banana plants (Hodgetts et al., 2015).

## 7 Challenges and future perspectives

Currently, there are three significant challenges associated with plant diagnostic tools. Concerns about the environmental impact and toxicity of synthetic nanomaterials, the urgency for faster data sharing and disease forecasting, and the durability of sensors in harsh conditions like extreme temperatures, intense sunlight, and heavy usage are paramount. Prior to field deployment, addressing safety concerns, particularly those related to hazardous nanoparticles like quantum dots (QDs), is crucial. Rigorous hazard assessment and oversight are necessary for nanosensors in contact with living plants or food to prevent toxic residues from entering the food chain and reaching consumers. The second obstacle pertains to the urgency for quicker reporting and real-time forecasting of disease outbreaks in agricultural settings. Advanced nanosensors are anticipated to be highly interconnected, facilitating nearly instantaneous monitoring. For instance, continuous tracking of plant volatile organic compound emissions offers more dynamic and precise data than sporadic measurements, thereby improving stress response monitoring. Finally, durable sensors capable of withstanding various environmental conditions, such as temperature fluctuations, humidity, and air pollution, are required before implementing these sensors in real-world agricultural settings. Further research is needed to develop innovative sensor materials, including environmentally resistant substrates with embedded nanoparticles.

## 8 Conclusion

One of the most significant global challenges is the substantial loss of agricultural production due to plant diseases. Plant diseases can greatly reduce crop yields, resulting in a substantial loss of resources and agricultural output. In order to develop effective strategies for disease diagnosis and mitigation, it is crucial to initially identify the prevailing plant diseases. Conventional approaches to identify plant pathogens, like scrutinizing infected tissue with a microscope or employing culture-based methods, are laborious and demand skilled personnel. However, modern techniques like biosensors offer a faster and more accessible means of disease identification. Increasing attention is being directed towards biosensors to detect phytopathogenic bacteria, with researchers striving to create portable handheld devices for swift, accurate, and site-specific detection. The convergence of biosensor technology and synthetic biology is a promising avenue of exploration for agricultural scientists.
